# Principles of a personalized approach in psychosocial interventions for cardiac surgery patients

**DOI:** 10.1192/j.eurpsy.2024.1269

**Published:** 2024-08-27

**Authors:** O. Nikolaeva, V. Babokin, N. Trofimov, S. S. Fakhraei

**Affiliations:** ^1^Department of Hospital Therapy, Ulianov Chuvash State University; ^2^Chuvash Republic Cardiology Clinic; ^3^Department of Surgical Diseases; ^4^Medical Faculty, Ulianov Chuvash State University, Cheboksary, Russian Federation

## Abstract

**Introduction:**

Cardiac surgery patients, former cardiac patients, face additional sources of stress connected with surgical intervention.

**Objectives:**

To devise the main principles of a personalized approach in psychosocial interventions for cardiac surgery patients.

**Methods:**

We have devised these principles based on the analysis of contemporary scientific literature and the operational experience of the Cardiology Clinic of the Chuvash Republic located in the city of Cheboksary.

**Results:**

A personalized approach in psychosocial interventions for cardiac surgery patients is used at all levels of medical support. It implies taking into consideration in every specific patient a unique correlation of their clinic-anamnestic peculiarities, clinic-psychological risk factors of the condition’s gravity and their psychological resources. At the same time, all the psychological interventions must focus on the personality and comply with the clinic specificity of the actual somatic and mental condition of the cardiac surgery patients. The underlying principles of the personalized approach in psychosocial interventions for cardiac surgery patientsinclude the principles of accessibility, openness, continuity, collaboration, integration, differentiation, variation, participation, awareness and prevention.

**Conclusions:**

Relying on the personalized approach in psychosocial interventions for cardiac surgery patientsallows working out a personalized treatment and rehabilitation course for an individual patient.

**Disclosure of Interest:**

None Declared

